# Therapeutic advances and future directions in *Helicobacter pylori* eradication

**DOI:** 10.3389/fmicb.2025.1652943

**Published:** 2025-12-03

**Authors:** Vidya Suresh, Amritavarshini Sreekumar, Anil Kumar, Shine Sadasivan, Raja Biswas, Lalitha Biswas

**Affiliations:** 1 Amrita School of Nanosciences and Molecular Medicine, Amrita Vishwa Vidyapeetham, Kochi, Kerala, India; 2 Department of Microbiology, Amrita Institute of Medical Sciences and Research Centre, Amrita Vishwa Vidyapeetham, Kochi, Kerala, India; 3 Department of Gastroenterology and Hepatology, Amrita Institute of Medical Sciences and Research Centre, Amrita Vishwa Vidyapeetham, Kochi, Kerala, India

**Keywords:** *Helicobacter pylori*, eradication therapy, antibiotic resistance, clinical trials, quadruple therapy, vonoprazan, probiotic adjuncts, phage therapy

## Abstract

*Helicobacter pylori (H. pylori)* is a globally prevalent pathogen associated with a spectrum of gastrointestinal diseases, including chronic gastritis, peptic ulcer disease, and gastric malignancies. Although clarithromycin-based triple therapy continues to be effective in certain geographic areas, increasing global resistance highlights the need for treatment approaches tailored to local antibiotic susceptibility patterns. Recent clinical trials have shifted toward optimized quadruple regimens, particularly bismuth- and vonoprazan-based therapies, and resistance-guided treatment. Novel agents such as Rifasutenizol (TNP-2198) are entering phase 3 evaluation and show activity against multidrug-resistant strains. Adjunctive and alternative strategies including probiotics, phytochemicals, phage-derived enzymes, and nanoparticle-based delivery demonstrate synergistic effects in improving eradication and reducing antibiotic-associated adverse events. Multi-strain probiotic formulations and plant-derived compounds such as curcumin, catechins, and quercetin consistently suppress *H. pylori* virulence pathways and gastric inflammation in clinical and preclinical studies. This review provides an overview of current evidence from clinical trials and emerging therapeutic innovations, emphasizing balanced integration of conventional and next-generation approaches for sustainable global *H. pylori* management.

## 1 Introduction

*Helicobacter pylori* (*H. pylori*) remains one of the most prevalent chronic bacterial infections globally, affecting an estimated 4.4 billion people (Öztekin et al., 2021). Its distribution shows marked geographic variation with prevalence exceeding 70% in Africa, Latin America, and Asia, while it remains considerably lower in North America, Oceania, and parts of Europe. These disparities reflect differences in sanitation, socioeconomic status, access to healthcare, and public health infrastructure (Zamani et al., 2018; Hooi et al., 2017). Despite a gradual decline in high-income regions due to improved hygiene and antibiotic use, transmission persists in developing countries, often acquired during early childhood (Salih, 2009). Classified as a Group 1 carcinogen by the WHO, *H. pylori* infection is a key risk factor for peptic ulcer disease and gastric cancer, contributing significantly to global morbidity and mortality (Reyes, 2023). The bacterium harbors several virulence factors, such as CagA, VacA, and urease, to disrupt epithelial integrity, evade immune responses, and sustain mucosal inflammation, which can progress to atrophic gastritis, intestinal metaplasia, and ultimately gastric cancer (Sirit and Peek, 2025). Transmission occurs primarily via the fecal-oral or oral-oral route, often during early childhood, with intrafamilial spread and environmental sources such as contaminated water playing key roles (Kayali et al., 2018).

Treatment of *H. pylori* infection has become increasingly complex due to growing antibiotic resistance, suboptimal patient compliance, and limited diagnostic capabilities in resource-limited settings. Standard triple therapy (STT), once the mainstay, now shows declining eradication rates often below the acceptable 80% threshold particularly in areas with high clarithromycin resistance (Nyssen et al., 2024). Reinfection, especially in high-prevalence regions, further complicates long-term disease management (Mladenova, 2021).

These challenges have necessitated a paradigm shift toward resistance-guided therapy and tailored treatment regimens. Quadruple therapies, non-bismuth combinations, and novel acid-suppressive agents such as potassium-competitive acid blockers (P-CABs) are being explored in clinical trials to overcome rising resistance (Rocha et al., 2025). In this review, we examine recent advancements in first- and second-line treatment strategies, emerging therapies, and ongoing clinical trials aimed at improving global management of *H. pylori*.

## 2 Evolution and current first-line regimens for *H. pylori* eradication

The therapeutic landscape for *H. pylori* infection has undergone a marked shift over the past 2 decades, largely in response to escalating antibiotic resistance, particularly to clarithromycin and levofloxacin (Hu et al., 2017). Once considered the cornerstone of eradication, clarithromycin-based standard triple therapy (STT), which includes a proton pump inhibitor (PPI), amoxicillin, and clarithromycin, is now widely discouraged. Most guidelines recommend its use only in regions where clarithromycin resistance is reliably below 15–20% (Nyssen et al., 2024; Prasertpetmanee et al., 2013). Declining global eradication rates, often below 80% in intention-to-treat (ITT) analyses, have prompted a transition toward more robust first-line options that offer greater efficacy in resistant settings (Nyssen et al., 2024). Among these, bismuth quadruple therapy (BQT), which includes a PPI, bismuth subcitrate, tetracycline, and metronidazole, has emerged as a first-line regimen of choice, especially in areas with moderate to high antibiotic resistance rates. Ten- to fourteen-day BQT regimens routinely achieved eradication rates close to 90% ITT. Shorter 10-day courses have also shown improved compliance and fewer side effects fewer adverse events (Yang et al., 2024).

Vonoprazan-based therapies have further transformed the treatment landscape. Vonoprazan, a novel P-CAB that enhances gastric acid suppression and improves antibiotic stability has emerged as a highly effective component of first-line *H. pylori* eradication regimens (Elsabaawy et al., 2024). Both vonoprazan-amoxicillin dual therapy and vonoprazan-amoxicillin-clarithromycin triple therapy have demonstrated superior or comparable efficacy to BQT in multiple randomized controlled trials. Dual therapy achieved eradication rates exceeding 90 % in per-protocol (PP) analyses, with fewer adverse events than BQT, however, these high success rates have been reported primarily in Asian populations (Qiu et al., 2024; Hu et al., 2025). The American College of Gastroenterology (ACG) recommends BQT for 14 days as the preferred first-line regimen in treatment-naïve patients when antibiotic susceptibility is unknown. For those without a penicillin allergy, empiric alternatives include vonoprazan-amoxicillin dual therapy for 14 days. Although vonoprazan triple therapy is approved as an empiric first-line option, its use is best reserved for patients infected with *H. pylori* strains confirmed to be sensitive to clarithromycin. These findings have positioned vonoprazan-containing regimens as preferred first-line options where available, especially in regions with high macrolide resistance outperforming both standard PPI-based triple therapy and some BQT (Liu L. et al., 2024).

## 3 Non-bismuth structured combination regimens

Non-bismuth structured combination regimens, also known as non-bismuth quadruple therapies, are multi-antibiotic treatment strategies designed to eradicate *H. pylori* without the use of bismuth salts. Sequential, concomitant, and hybrid regimens represent structured approaches developed to enhance eradication efficacy, particularly in the presence of single-drug resistance.

Sequential therapy follows a two-phase protocol and involves 5 days of PPI + amoxicillin, followed by 5 days of PPI + clarithromycin + metronidazole. It was designed to reduce the impact of macrolide resistance, but comparative advantages over concomitant regimens have been inconsistent across regions. While concomitant therapy administers PPI, amoxicillin, clarithromycin, and metronidazole, simultaneously for 10–14 days. It can achieve high eradication rates even with single antibiotic resistance, but it increases pill burden and exposure to multiple antibiotics. Hybrid therapy, combines elements of both sequential and concomitant regimens, starts with dual therapy (PPI + amoxicillin) for 7 days, followed by quadruple therapy in the latter half and has shown eradication rates up to 93.9% with improved compliance due to reduced pill burden (Song and Zhou, 2016). Among these, concomitant therapy has shown superior eradication rates (up to 89%), particularly in the presence of dual antibiotic resistance (Wu et al., 2010; Hsu et al., 2015). High-dose dual therapy with PPI and amoxicillin has also demonstrated promise, particularly in settings where resistance to clarithromycin and levofloxacin was high. This regimen has achieved ITT eradication rates around 89%. Although the 2024 ACG guidelines do not prioritize these regimens for treatment-naïve patients, they remain valuable alternatives where bismuth is unavailable or poorly tolerated (Song and Zhou, 2016). The choice of first-line therapy should be guided by local resistance patterns, patient history, and drug availability. Confirming eradication post-treatment remains a critical component of effective management.

## 4 Second-line and salvage therapies

When first-line treatments fail, second-line and salvage therapies become necessary. These often consist of alternative antibiotic combinations or repeated quadruple therapy, guided by antibiotic susceptibility testing when feasible. Optimized BQT remains a dependable second-line option for patients who have not previously received BQT, particularly when antibiotic susceptibility is unknown or following failure of STT. Its broad-spectrum activity and minimal cross-resistance with clarithromycin and levofloxacin support its continued use in such settings. Levofloxacin-based regimens and rifabutin-containing combinations are effective salvage options in many settings; rifabutin is particularly useful when resistance to macrolides and fluoroquinolones is present. Levofloxacin-based triple therapy (PPI, levofloxacin, amoxicillin) is a common second-line option. Its use is becoming increasingly limited due to the growing prevalence of levofloxacin resistance with eradication rates significantly declining in regions where resistance exceeded > 20% (Kim and Chung, 2020). However, tetracycline-levofloxacin quadruple therapy, which combines a PPI, bismuth, tetracycline, and levofloxacin, has shown higher efficacy and achieved eradication rates up to 98% in recent studies. Sequential levofloxacin therapy offered similar efficacy (90–93%) with fewer pills (Shih et al., 2023; Abdulqader et al., 2025). Levofloxacin-rifaximin quadruple therapy achieved 90.6% eradication with fewer side effects (15 vs. 28%) than hybrids (82.2%). Levofloxacin regimens had fewer discontinuations (3 vs. 8%) and mild GI effects (15–28%). However, when levofloxacin resistance exceeds 30%, treatment efficacy drops significantly, reaching only 60–70% (Kim and Chung, 2020). Rifabutin-based therapy has emerged as a promising salvage option. Low resistance rates, estimated at around 1%, and potent antimicrobial activity make rifabutin an effective agent in patients with multiple prior treatment failures (Nyssen et al., 2022; Graham et al., 2020). Though comparative studies are limited, rifabutin triple therapy (PPI, amoxicillin and rifabutin) typically achieved eradication rates between 73 and 87%, which was higher than BQT (67%). Extending therapy to 14 days further enhanced efficacy. Some studies report eradication rates over 90% when rifabutin is combined with levofloxacin or administered after tailored antibiotic selection (Gisbert, 2020). A standard 10–12 day rifabutin triple regimen (esomeprazole, amoxicillin, rifabutin) was effective against clarithromycin- and levofloxacin-resistant strains (Nyssen et al., 2022; Graham et al., 2020; Perri et al., 2001). Emerging salvage regimens such as sitafloxacin-based combinations are gaining attention in regions with low macrolide resistance, along with furazolidone-containing therapies. However, their wider application remains limited due to safety concerns and restricted availability.

**FIGURE 1:**
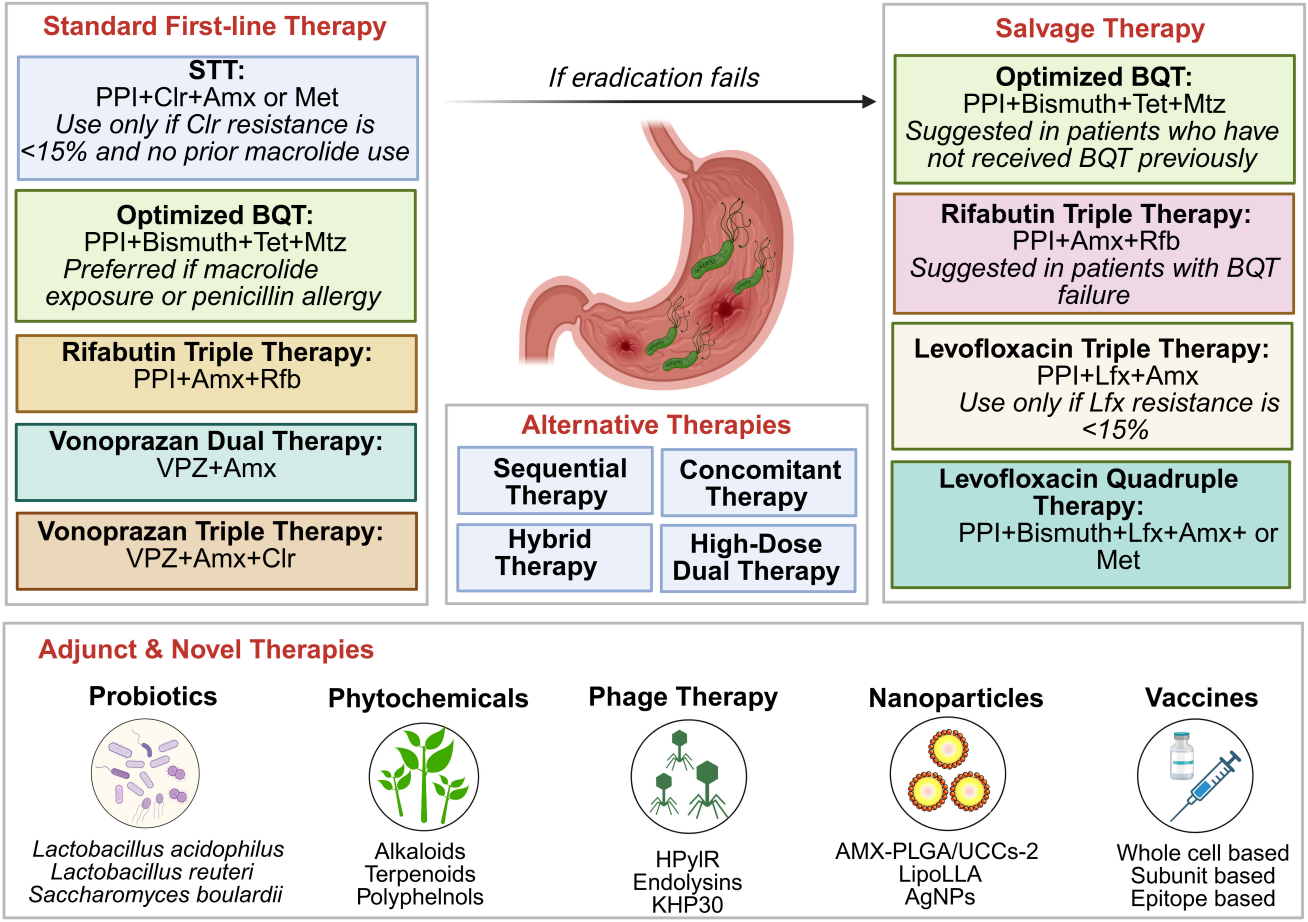
Algorithm for the management of *Helicobacter pylori* infection. Standard first-line therapy options include standard triple therapy (STT), optimized bismuth quadruple therapy (BQT), rifabutin triple therapy (2024 ACG guidelines listed RTT as a suitable empiric alternative for first-line use in patients without penicillin allergy), and vonoprazan-based regimens. In cases of treatment failure, salvage therapy regimens such as optimized BQT, rifabutin triple therapy, and levofloxacin-based therapies are recommended depending on prior exposure and resistance patterns. Alternative therapies include sequential, concomitant, hybrid, and high-dose dual therapies. Adjunct and novel strategies under investigation include probiotics, phytochemicals, phage therapy, nanotherapy, and vaccines aimed at enhancing eradication rates and reducing resistance. PPI, proton pump inhibitor; ClR, clarithromycin; Amx, amoxicillin; Met, metronidazole; Tet, tetracycline; Mtz, metronidazole; Rfb, rifabutin; VPZ, vonoprazan; Lfx, levofloxacin; HPyIR, *Helicobacter pylori* infecting recombinant phage; KHP30, a specific endolysin/phage component targeting *H. pylori*; AMX-PLGA/UCCs-2, amoxicillin-loaded poly(lactic-co-glycolic acid) ureido-conjugated chitosan derived nanoparticles; LipoLLA, liposomes loaded with linolenic acid nanoparticles; AgNPs, silver nanoparticles; whole cell based, vaccines derived from inactivated or attenuated *H. pylori* cells; subunit based and epitope based, vaccines targeting specific antigenic such as CagA, VacA, BabA, SabA, HopQ, OipA, Hsp60, and NapA of *H. pylori.*

For patients with confirmed penicillin allergy, BQT is recommended as the primary first-line option. In regions with low macrolide resistance, vonoprazan-based therapy with clarithromycin and metronidazole may be considered. Levofloxacin-containing regimens can serve as alternative choices when fluoroquinolone resistance is low and prior antibiotic exposure has been assessed. Amoxicillin-containing regimens should be avoided in this population. These therapeutic regimens, illustrated in Figure 1, must be tailored based on culture or molecular susceptibility testing and individual treatment history and regional resistance data to improve eradication rates and minimize adverse effects (Figure 1).

## 5 Global clinical evidence and population-based strategies

In addition to individual therapeutic innovations, growing evidence from large-scale clinical trials and population-based interventions has provided valuable insights into the real-world impact of *H. pylori* eradication strategies, particularly in reducing gastric cancer risk and improving public health outcomes.

### 5.1 Large-scale and public health trials

Large-scale clinical trials and public health strategies have underscored the importance of widespread screening and targeted eradication in reducing the burden of *H. pylori* infection and its complications, particularly gastric cancer and peptic ulcer disease. The HEAT trial, involving over 5,000 participants in the UK, demonstrated a significant reduction in peptic ulcer bleeding following *H. pylori* eradication, especially in the first 2.5 years post-treatment (Hawkey et al., 2022). Similarly, the MITS trial in China, one of the largest community-based interventions (180,284 participants), showed that 10-day quadruple therapy reduced infection incidence and mortality in the 25–45 age group (Liu and Li, 2025). These studies support integrating eradication therapy into routine care for high-risk populations. The LEGACy Consortium, encompassing multiple countries, confirmed the superiority of quadruple regimens over STT, particularly in antibiotic-resistant regions. Both BQT (91.3%) and concomitant quadruple therapy (88.7%) outperformed STT (75.2%), strengthening the case for updating national guidelines based on regional resistance data (Medel-Jara et al., 2024). In addition to these trials, public health initiatives advocate for screen-and-treat programs, especially in high-incidence regions (Leung et al., 2008; Pasechnikov et al., 2014). These approaches, if implemented before the onset of precancerous lesions, can be cost-effective and reduce gastric cancer incidence by up to 53% (Liu Z. et al., 2024). These programs are most effective when integrated with existing cancer screening frameworks, healthcare provider training, and public awareness campaigns. Probiotic adjuncts like *Limosilactobacillus reuteri* DSM 17648 have also been tested in multi-country trials, highlighting their role in enhancing tolerability and possibly improving eradication outcomes (Dargenio et al., 2021; Ismail et al., 2023).

### 5.2 Therapeutic innovation in clinical trials

Recent meta-analyses and clinical trial registries underscore significant advancements in *H. pylori* eradication strategies. Between 2015 and 2024, more than 100 clinical trials focusing on *Helicobacter pylori* eradication were registered across major trial registries, including ClinicalTrials.gov, UMIN, and WHO ICTRP, reflecting a concerted global effort to improve eradication efficacy, minimize side effects and address growing challenge of antibiotic resistance. Asia, especially China, Japan, and South Korea, accounted for a large proportion of clinical trials, reflecting both the high disease burden and proactive screening initiatives in these regions (Cho and Jin, 2022). These trials were predominantly interventional, randomized, and multicentric, with patient enrolment ranging from under 100 to more than 2,000 participants. While adult populations were the most studied, a growing number of trials addressed pediatric, elderly, and comorbid cohorts. These trials explored a broad spectrum of therapeutic strategies, with particular emphasis on optimizing efficacy in diverse patient subgroups and resistance settings. Nearly half of all trials investigated quadruple regimens, particularly bismuth-based and concomitant therapies. A large Taiwanese study (NCT04527055) demonstrated that 10-day and 14-day BQT achieved comparable eradication rates (∼92–93% ITT; 98–99% PP) with less serious adverse events offering better compliance (Yang et al., 2024). Another meta-analysis of also confirmed that quadruple therapies consistently outperform triple regimens, particularly in regions with high clarithromycin resistance (Venerito et al., 2013). Several trials assessed variations of quadruple therapy, including comparisons of different PPIs and bismuth formulations. For example, studies evaluating lansoprazole- vs. rabeprazole-containing quadruple regimens showed minor differences in eradication rates but notable variation in tolerability, particularly in elderly patients (Kan et al., 2020). A comprehensive network meta-analysis of 55 studies identified combinations including rabeprazole, bismuth, furazolidone, and tetracycline as the most effective, while lansoprazole-based regimens had the best safety profile (Wei et al., 2025). Hybrid and sequential therapies also featured prominently, with variable success depending on local resistance profiles.

Several multicenter trials, including NCT04167670, NCT04527055, NCT01505127, ChiCTR2300070475, ChiCTR2100045778, UMIN000016336, and EudraCT 2019-002668-28, have confirmed the consistent superiority of vonoprazan-containing regimens across first- and second-line settings. These regimens are increasingly being favored in regions with high resistance to clarithromycin (Sue et al., 2019; Murakami et al., 2016; Zhou et al., 2024). Most importantly, vonoprazan-based dual and triple therapies consistently demonstrate high eradication rates, rivaling or surpassing BQT with fewer adverse effects even in areas of high levofloxacin and clarithromycin resistance (Tungtrongchitr et al., 2024). For patients failing first-line treatment, second-line options including levofloxacin-based and rifabutin-containing regimens were evaluated in multiple trials (NCT03198507, NCT04652284). Levofloxacin combined with tetracycline or bismuth improved outcomes in resistant infections, while rifabutin-based triple therapy achieved success rates up to 91%, offering a viable salvage strategy in multi-drug-resistant cases (NCT06351891) (Wang et al., 2025). Some studies explored sequential and hybrid therapies tailored to local resistance profiles. These approaches include modifications in the timing and combination of antibiotic administration, as well as the use of alternative agents such as furazolidone and minocycline to address resistance to metronidazole or clarithromycin. Although often tested in small patient populations, these regimens have demonstrated eradication rates ranging from 75 to 92% and were especially promising in South Asian and Middle Eastern settings (Cardos et al., 2021; Hu et al., 2017; Zhang et al., 2021). Second-line and rescue therapy trials included further exploration of levofloxacin- and rifabutin-based combinations (Gisbert, 2020). Some studies evaluated high-dose dual therapies such as high-dose amoxicillin with PPI or P-CAB administered over shorter durations and were aimed at simplifying regimens for improved patient compliance (Yang et al., 2025). These strategies were particularly relevant in patients with multiple prior treatment failures or penicillin allergies (Rokkas et al., 2025). While these trials provide important insights into regimen refinement and patient-centered outcomes, adjunctive interventions have also gained increasing attention for their potential to support eradication and reduce treatment-related side effects. Some trials have investigated the use of mucosal protectants like rebamipide and sucralfate in combination with standard therapies, aiming to promote mucosal healing and reduce adverse events. In a recent trial (NCT05857163), Rifasutenizol-based triple therapy achieved high *H. pylori* eradication rates with efficacy comparable to standard bismuth–clarithromycin–based regimens, highlighting its potential as a novel dual-mechanism first-line therapy. A selection of recent clinical trials is summarized in Table 1, highlighting key therapies, durations, eradication outcomes, and resistance considerations (Table 1).

**TABLE 1:** Summary of selected clinical trials on H. pylori eradication.

No.	Clinical trial no.	Therapy type	Eradication rate	Overall implication	Resistance-specific efficacy	Adverse effects	References
1	NCT03198507	RHB-105 vs. high-dose PPI-AMO (14d)	RHB-105: 83.8–90.3%, comparator: 57.7–64.7%	RHB-105 superior, effective in resistant strains	RHB-105 offers high eradication rates and is effective even against despite CLR/MET resistance strains	Generally, well tolerated. Most common AEs (≥5%): Diarrhea (10.1%), Headache (7.5%), Nausea (4.8%)	(Graham et al., 2020; Kalfus et al., 2020)
2	NCT04167670	Vonoprazan dual, vonoprazan triple, lansoprazole triple	Triple: 80.8%, dual: 77.2%, lansoprazole: 68.5%; In CLR resistant infections: dual > triple > lansoprazole	Vonoprazan regimens were superior overall and in resistant strains; good safety	In CLR-resistant infections vonoprazan therapies were significantly more effective: vonoprazan triple: 65.8% vonoprazan dual: 69.6% lansoprazole triple: 31.9%	The frequency of treatment-emergent adverse events was similar across all regimens	(Chey et al., 2022)
3	EudraCT number: NCT05718609	Tailored bismuth quadruple therapy (BQT): rabeprazole + colloidal bismuth + amoxicillin + clarithromycin or furazolidone (based on fecal resistance profiling)	- ITT: 87.44% (fecal-tailored) vs. 82.00% (empirical); - mITT: 89.23 vs. 82.41%; - PP: 94.57 vs. 85.86%; (superiority in PP: +7.07%, 95% CI: 0.90–13.25%)	Fecal-based tailored BQT is noninferior to empirical BQT and more effective in PP analysis; supports use as first-line therapy in resistant settings.	Effectively substitutes furazolidone for clarithromycin when resistance is detected via fecal molecular testing, avoiding invasive endoscopy.	Adverse events and compliance rates were similar across groups; no significant increase in side effects with tailored regimens.	(Yu et al., 2025)
4	ClinicalTrials.gov ID: NCT06351891	Cef-Tet BQT: tegoprazan + bismuth potassium citrate + cefuroxime + tetracycline; comparator: cefuroxime-levofloxacin BQT (Cef-Lev BQT)	- ITT: 90.32% (Cef-Tet) vs. 81.45% (Cef-Lev), *p* = 0.045; - mITT: 91.80% vs. 83.47%, *p* = 0.048; - PP: 92.37% vs. 85.34%, *p* = 0.087; (Noninferior in all)	Cef-Tet BQT is a safe, effective, and noninferior first-line option for penicillin-allergic patients; it avoids levofloxacin-related resistance concerns.	Avoids fluoroquinolone resistance risk by excluding levofloxacin; tetracycline and cefuroxime retain activity in penicillin-allergic settings.	Similar incidence of adverse events: 21.77% (Cef-Tet) vs. 24.19% (Cef-Lev); high compliance in both groups (96.77 vs. 95.97%). No significant safety concerns.	(Wang et al., 2025)
5	ClinicalTrials.gov ID: NCT05431075	Bismuth quadruple therapy with tetracycline either 3x/day (TET-T) or 4x/day (TET-F), plus esomeprazole, bismuth, and amoxicillin	ITT: 91.63% (TET-T) vs. 90.15% (TET-F); PP: 95.34 vs. 95.72%; (_TET-T noninferior, *p* < 0.001)	TET-T is equally effective as TET-F and offers better tolerability; supports simplified 3x daily tetracycline dosing in BQT.	Not resistance-guided, but effective empirical first-line option where tetracycline resistance is low or absent.	Lower in TET-T group: 21.61 vs. 31.63% (*p* = 0.024); compliance similar across both groups	(Ding et al., 2024)
6	Chinese clinical trials registry (registration ID: ChiCTR2000033717	MOEB: minocycline, ornidazole, esomeprazole, bismuth vs. ACEB: amoxicillin, clarithromycin, esomeprazole, bismuth potassium citrate	MOEB: 93.2% (PP), 78.5% (ITT) ACEB: 82.5% (PP), 72.8% (ITT)	MOEB is more effective and better tolerated than ACEB, and is a viable first-line empirical therapy.	Not directly tested, but MOEB likely circumvents clarithromycin resistance through use of minocycline and ornidazole.	Lower with MOEB: 19.3 vs. 33.8% (*p* = 0.0019); better safety profile	(Lin et al., 2025)
7	ClinicalTrials.gov ID: NCT05649709	VA dual therapy [vonoprazan 20 mg BID + amoxicillin 2 g/day (LVA) or 3 g/day (HVA)]	LVA: 85.3% (ITT), 88.8% (PP) HVA: 86.5% (ITT), 92.4% (PP)	LVA is non-inferior to HVA and equally effective as first-line therapy; high-dose amoxicillin is not required for efficacy.	No amoxicillin resistance observed; both regimens effective without prior susceptibility testing.	Lower in LVA (12%) vs. HVA (17%); both well tolerated	(Hu et al., 2025)
8	ClinicalTrials.gov ID: NCT06250634	High-dose dual therapy (HDDT) with either: • esomeprazole + amoxicillin (EA-dual); • vonoprazan + amoxicillin (VA-dual). compared to: • B-quadruple (EA + clarithromycin + bismuth); • VAC (vonoprazan + amoxicillin + clarithromycin).	- EA-dual: ITT 70.59%, mITT 92.86%, PP 93.94%; - B-quadruple: ITT 83.49%, mITT and PP 98.38%; - VA-dual: ITT 84.15%, mITT 96.25%, PP 96.75%; - VAC: ITT 83.15%, mITT 92.73%, PP 93.75%.	VA-dual HDDT is non-inferior to VAC and may serve as a cost-effective, first-line regimen. EA-dual is non-inferior to B-quadruple in mITT and PP analyses but had lower ITT eradication.	Prior antibiotic use (within 2 years) negatively impacted eradication success, highlighting resistance as a relevant factor.	No significant differences in adverse events, compliance, or symptom relief among groups; VA therapy had the lowest cost	(Yang et al., 2025)
9	Chinese clinical trial registry no: ChiCTR2100055101	VAS regimen: vonoprazan + amoxicillin + *Saccharomyces boulardii* for 10 days; compared to ECAB: esomeprazole, clarithromycin, amoxicillin, bismuth (14-day quadruple)	- VAS: ITT 87.3%, PP 87.3%; - ECAB: ITT 88.9%, PP 91.8%.	VAS regimen was non-inferior to ECAB, with fewer adverse events and lower cost; particularly beneficial for patients with low body surface area (BSA) and non-smokers.	Not directly measured, but non-smoking status and low BSA were associated with better outcomes, suggesting host factors influenced success more than antibiotic resistance.	Fewer in VAS group compared to ECAB group; VAS had a better safety profile.	(Yu et al., 2024)
10	ClinicalTrial.gov ID: NCT05614934	- VDT: vonoprazan + amoxicillin/clavulanate (dual); - VTT: vonoprazan + amoxicillin/clavulanate + clarithromycin (triple); - STT: PPI-based standard triple therapy.	- STT: 70%; -VDT: 76.2%; - VTT: 79.2% (per-protocol analysis, *p* = 0.777).	All regimens showed suboptimal efficacy, underscoring the need for dose/frequency optimization and new regimen development. VDT had a better safety profile.	Not directly assessed; however, the limited cure rates suggest potential impact of antimicrobial resistance, especially to clarithromycin.	VDT had the best safety profile among the three, though the differences were not statistically significant	(Shekeban et al., 2025)
11	ClinicalTrial.gov ID: NCT05857163	RTT: rifasutenizol 400 mg + amoxicillin 1 g + rabeprazole 20 mg, twice daily for 14 days vs. BCTT: bismuth-clarithromycin triple therapy	RTT: 92.0% (95% CI 88.7–94.6); BCTT: 87.9% (95% CI 83.9–91.1); absolute difference: 4.2% (95% CI –0.3 to 8.8); non-inferior to BCTT.	RTT showed high eradication efficacy and was non-inferior to standard regimens, representing a novel dual-action antimicrobial and a promising first-line therapy for *H. pylori* infection.	All *H. pylori* isolates were susceptible to rifasutenizol, and RTT maintained high eradication rates despite high clarithromycin and metronidazole resistance, highlighting its potential against resistant strains.	Well tolerated. Most common AEs (≥5%) in RTT group: diarrhea (7%), nausea (6%), dizziness (6%). No serious treatment-related adverse events reported	(Song et al., 2025)

RHB-105, Rifabutin-Based Triple Therapy (AMO+omeprazole+ rifabutin); AMO, Amoxicillin; PPI, Proton pump inhibitor; STT, Standard Triple Therapy (PPI+CLR+AMO); CLR, Clarithromycin; MET, Metronidazole; LEV, Levofloxacin; BQT, Bismuth Quadruple Therapy; ITT, Intention-to-treat analysis; AEs, Adverse events; PP, Per-protocol analysis; mITT, Modified intention-to-treat analysis; Cef, Cefuroxime; Tet, Tetracycline; TET-T, Tetracycline 500 mg three times daily; TET-F, Tetracycline 500 mg four times daily; MOEB, Minocycline, Ornidazole, Esomeprazole, and Bismuth; ACEB, Amoxicillin, Clarithromycin, Esomeprazole, and Bismuth potassium citrate; VA, Vonoprazan and amoxicillin; LVA, Vonoprazan (20 mg twice a day) with low-dose amoxicillin (1 g twice a day); HVA: Vonoprazan (20 mg twice a day) with high-dose amoxicillin (1 g three times a day); HDDT, High-dose dual therapy; EA: Esomeprazole + Amoxicillin; VAC, Vonoprazan + Amoxicillin + Clarithromycin; VAS, Vonoprazan + Amoxicillin + *Saccharomyces boulardii*; ECAB, Esomeprazole, Clarithromycin, Amoxicillin, and Bismuth; VDT, Vonoprazan Dual Therapy (Vonoprazan 20 mg twice daily + amoxicillin/clavulanate 875 mg/125 three times daily); VTT, Vonoprazan Triple Therapy (Vonoprazan 20 mg twice daily + amoxicillin/clavulanate 875 mg/125 three times daily + clarithromycin 500 mg twice daily); RTT, Rifasutenizol-based triple therapy (Rifasutenizol 400 mg, amoxicillin 1 g, and rabeprazole 20 mg, all twice day for 14 days); BCTT, Bismuth + clarithromycin-based triple therapy (Bismuth potassium citrate 240 mg + clarithromycin 500 mg + amoxicillin 1 g, and rabeprazole 20 mg, all twice a day for 14 days).

### 5.3 Trends in clinical trial design and scope

European and North American guidelines and trials often emphasized advanced acid suppression strategies and resistance-guided therapy, while trails conducted across India, Turkey, Egypt, and Brazil emphasized the need for cost-effectiveness and simplified regimens, underscoring the real-world applicability of findings in resource-constrained settings.

#### 5.3.1 Personalized and susceptibility-guided therapies

Resistance-guided therapy using either genotypic or phenotypic methods was incorporated in approximately 20–30% of trials, with several employing molecular diagnostics to detect clarithromycin and levofloxacin resistance mutations prior to treatment initiation (Ng et al., 2023). PCR-based assays for the detection of clarithromycin and levofloxacin resistance mutations and non-invasive tools such as the string test combined with qPCR have shown potential to guide therapy selection and improve eradication rates, especially in high-resistance settings. Tailored regimens based on these approaches have achieved eradication rates of 86–91%, highlighting the potential of personalized treatment strategies in *H. pylori* management (Cui et al., 2024). Although these strategies offer targeted treatment advantages, empirical use of optimized quadruple therapy remains comparably effective in many cases.

#### 5.3.2 Novel and adjunctive therapies for *H. pylori* infection

With rising antibiotic resistance and treatment failure rates, novel and adjunctive therapies are being explored to enhance the efficacy and tolerability of *H. pylori* eradication regimens. These include probiotics, phage therapy, engineered enzymes, phytochemicals, nanoparticles and immunomodulatory strategies.

##### 5.3.2.1 Probiotic adjuncts

Probiotics are increasingly recognized as valuable adjuncts in *Helicobacter pylori* eradication therapy due to their ability to restore gut microbiota balance, reduce antibiotic-associated side effects, and enhance treatment adherence (Bai et al., 2022; Goderska et al., 2018; Meliţ et al., 2022). Current consensus, supported by the Maastricht V and VI reports and World Gastroenterology Organization guidelines, recommends probiotics primarily as supportive agents to reduce treatment-related gastrointestinal adverse events and modulate gastric inflammation rather than as standalone therapies. Although some studies have reported direct antibacterial or anti-adhesive effects against *H. pylori*, their clinical eradication efficacy as monotherapy remains limited and inconsistent (Malfertheiner et al., 2017; Katelaris et al., 2023).

The beneficial effects of probiotics are largely attributed to their anti-inflammatory and microbiota-stabilizing actions. Strains such as *Lactobacillus rhamnosus* GG, *L. reuteri* DSM 17938, *Bifidobacterium lactis*, and *Saccharomyces boulardii* reduce mucosal inflammation by downregulating pro-inflammatory cytokines (IL-8, TNF-α) and enhancing mucosal immunity via increased secretory IgA (Goderska et al., 2018; Sousa et al., 2022). These effects lessen gastric irritation and mitigate antibiotic-induced dysbiosis, improving patient comfort and compliance during eradication therapy. Their direct bactericidal activity, mediated through competitive inhibition of *H. pylori* adhesion and production of organic acids, hydrogen peroxide, and bacteriocins, is strain-dependent and modest in clinical settings (Keikha and Karbalaei, 2021; Yu et al., 2025).

Meta-analyses and controlled trials consistently show that probiotic supplementation enhances both tolerability and eradication rates. Across pooled datasets of over 40 RCTs, eradication success improved by 10–15% (RR/OR 1.12–1.68), with significant reductions in diarrhea, nausea, and other gastrointestinal adverse effects (Zhang et al., 2015; Wang et al., 2023; Dang et al., 2014). Strain-level evidence supports this trend: *L. reuteri* DSM 17648 increased eradication from 68.9 to over 90% and reduced abdominal discomfort (Ismail et al., 2023). *Bifidobacterium animalis* subsp. *lactis* B94 improved eradication (86.8 vs. 70.8%, *p* = 0.025) and decreased diarrhea-related non-compliance, while *Saccharomyces boulardii* showed similar benefits with an 86.7% eradication rate and better overall tolerability (Viazis et al., 2022; Yu-Hao et al., 2025). Among multi-strain products, *Lacticaseibacillus rhamnosus* R0011 and *L. helveticus* R0052 (Lacidofil^®^) enhanced eradication from 75.0 to 90.9% and significantly reduced bloating, nausea, and bitter taste (Kiattiweerasak et al., 2023). A four-strain formula (*L. acidophilus, L. plantarum, B. lactis*, and *S. boulardii*) achieved 92.0% eradication vs. 86.8% with standard therapy and decreased new or worsened symptoms from 50.7 to 17.0% (Viazis et al., 2022). These findings suggest that combining complementary strains maximizes efficacy by uniting mechanisms such as *H. pylori* antagonism, restoration of gut eubiosis, and mitigation of antibiotic-associated dysbiosis.

Delivery systems are critical determinants of probiotic performance. Conventional oral formulations often lose viability in acidic gastric conditions, prompting the development of advanced encapsulation approaches. Microencapsulation in alginate or bilayer alginate/Eudragit matrices, mucoadhesive polymers, and enteric coatings improve gastric survival and targeted mucosal release, prolonging retention and enhancing both anti-inflammatory and anti-adhesive actions (Meliţ et al., 2022). Innovative co-formulations combining probiotics with prebiotics (synbiotics) or low-dose antibiotics are being explored to enhance therapeutic synergy and restore post-eradication microbial balance. Synbiotic preparations typically couple *L. acidophilus, B. lactis* Bb12, or *L. rhamnosus* GG with fermentable prebiotics such as inulin or fructooligosaccharides (FOS), which support probiotic persistence in the gastric and intestinal mucosa. Recent clinical trials (NCT06499649, NCT04527055) are evaluating such synbiotic-enhanced regimens for their ability to sustain eradication and preserve microbial diversity.

In summary, probiotics complement standard *H. pylori* therapy by improving tolerance, modulating gastric inflammation, and modestly increasing eradication efficacy. Their success depends on strain selection, formulation, and duration of administration. Further research should focus on defining optimal strain combinations, standardizing doses, and establishing their role in post-eradication microbiome recovery.

##### 5.3.2.2 Bacteriophage therapy

Phage therapy represents a promising targeted strategy for *H. pylori* eradication, particularly in the context of rising antibiotic resistance. While most *H. pylori*-infecting phages identified to date are temperate and not ideal for therapeutic use, efforts are underway to engineer strictly lytic variants or develop safer phage-derived alternatives such as endolysins (Abdel-Haliem and Askora, 2013). The phage HPy1R shows promise due to its gastric stability and ability to suppress *H. pylori in vitro*. However, its temperate nature necessitates genetic modification to create strictly lytic variants for safe clinical use (Ferreira et al., 2022). Phage-derived enzymes, such as endolysins, offer an alternative therapeutic option by enzymatically degrading peptidoglycan and minimizing concerns associated with whole-phage therapy, including lysogeny and immune activation (Abdelrahman et al., 2021). Genomic studies have identified multiple prophages within *H. pylori* that were linked to virulence and resistance, highlighting both therapeutic opportunities and biosafety challenges (Ferreira et al., 2025). Despite its potential, phage therapy for *H. pylori* faces key hurdles, including the need for clinical validation, effective gastric delivery systems, and resistance prevention strategies. Several clinical trials are currently underway to assess efficacy. Future research should optimize formulations, evaluate long-term safety, and ensure therapeutic stability for clinical use.

##### 5.3.2.3 Phytochemicals and plant-based compounds

**FIGURE 2:**
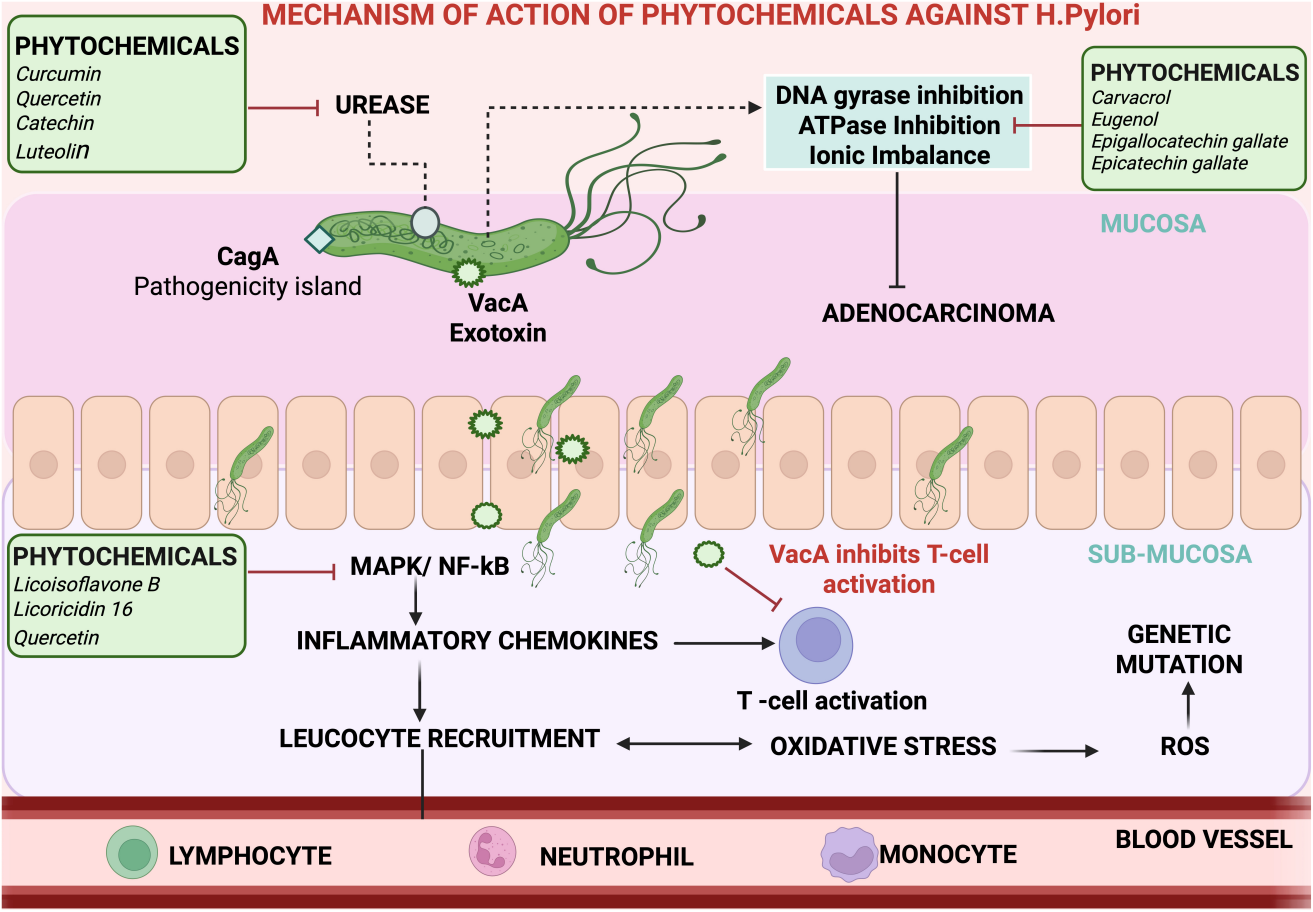
Mechanism of action of phytochemicals against *H. pylori.* Phytochemicals inhibit key virulence mechanisms of *H. pylori* by targeting urease, DNA gyrase, and ATPase activity, leading to impaired colonization and ionic imbalance. Compounds such as curcumin, quercetin, catechin, and luteolin suppress MAPK/NF-κB–mediated inflammation and oxidative stress, reducing leukocyte recruitment. Agents like carvacrol, eugenol, and epigallocatechin gallate further disrupt bacterial viability and modulate host immune responses, collectively mitigating VacA-mediated T-cell inhibition and epithelial injury that contribute to gastric carcinogenesis.

Phytochemicals and plant-derived compounds offer promising adjunctive benefits in *H. pylori* eradication therapy through their antibacterial, anti-inflammatory, antioxidant and resistance-modulating properties (Liu M. et al., 2024). Their mechanisms of action include inhibition of urease and DNA gyrase activity, disruption of bacterial adhesion and biofilm formation, attenuation of virulence factors such as VacA and CagA, and suppression of host inflammatory pathways like NF-κB and IL-8 signaling (Abou Baker, 2020; Meliţ et al., 2022). These multifaceted actions make them potential complements to conventional antibiotic regimens, especially in the context of rising resistance. Extracts from *Glycyrrhiza glabra* (licoisoflavone B and licoricidin 16 flavonoids; MIC: 6.25-50 μg/mL) *Cistus laurifolius (quercetin 3,7-dimethyl ether, kaempferol 3,7-dimethyl ether;* MIC: 3.9–62.5 μg/mL) curcumin (MIC: 5–50 μg/mL), 6-Gingerol (MIC: 40 μg/mL), and green tea (Epigallocatechin gallate, epicatechin gallate, epigallocatechin; MIC: 8–256 μg/mL) have demonstrated activity against both susceptible and resistant strains (Ustün et al., 2006; Tungmunnithum et al., 2018; Sathianarayanan et al., 2022; Wang, 2014; Asha et al., 2013). Terpenoids like carvacrol disrupt bacterial membranes (MIC μg/mL: 128 for *H. pylori* ATCC 43504), while polyphenols and flavonoids such as quercetin, catechin, and luteolin inhibit urease and DNA gyrase act at MIC values of ∼16–32, 64–128 and 32–64 μg/mL, respectively (Huang et al., 2024). Alkaloids like coptisine also impair urease activity, reducing bacterial survival in acidic environments (Huang et al., 2024; Figure 2). Recent investigations further highlight how formulation advancements enhance phytochemical efficacy. *Aloe vera* gel integrated with chitosan nanoparticles exhibited improved antibacterial, antioxidant, and anti-inflammatory properties compared to crude extracts, achieving inhibition zones of 28–30 mm and MIC/MBC values of 3.9/7.8 μg/mL (Yahya et al., 2022). Similarly, *Acacia nilotica* flower extract displayed significant anti-*H. pylori* effects with a 31 mm inhibition zone and MIC/MBC values of 7.8/15.6 μg/mL, attributed to high levels of ferulic acid, quercetin, and rutin (Al-Rajhi et al., 2023b). Moist heat-treated laurel (*Laurus nobilis* L.) leaf extract yielded enhanced phenolic content and strong anti-*H. pylori* activity, achieving 93.7% biofilm inhibition and an IC50 of 34.17 μg/mL for urease inhibition (Al-Rajhi et al., 2023a). In a similar approach, moist-heat treatment of rosemary extract was found to improve its antimicrobial potential, with an inhibition zone of 29.5 mm compared to 22 mm for untreated extract and MIC/MBC values of 3.9/7.8 μg/mL, this was linked to elevated rosmarinic and ellagic acid content (Bakri et al., 2024). Additionally, nanoformulations such as rutin nanocrystals (RNCs) exhibited improved solubility and bactericidal activity, producing an inhibition zone of 22.67 mm compared to 18 mm for rutin and a lower MIC/MBC of 7.8 μg/mL (Qanash et al., 2023). RNCs also achieved higher biofilm inhibition (92.12%) and stronger urease suppression (IC50 = 6.85 μg/mL), suggesting nanosizing as an effective enhancement strategy.

Many phytochemicals have also been found to modulate the host inflammatory responses by downregulating IL-8, TNF-α, and COX-2 expression, thereby mitigating gastric mucosal injury. Antioxidant-rich compounds such as curcumin and resveratrol also reduce oxidative stress, which contributes to mucosal healing and may enhance the success of eradication therapy (Abou Baker, 2020). Moreover, several polyphenols particularly catechins, resveratrol, and quercetin exhibit synergistic interactions with clarithromycin and metronidazole, decreasing the MICs of these antibiotics in resistant isolates (Meliţ et al., 2022). Advances in the biomaterial-based delivery systems have further expanded the therapeutic potential of plant-derived agents. Encapsulation of curcumin, catechins, or quercetin in chitosan, alginate, or PLGA nanoparticles enhances solubility, stability, and mucosal penetration while providing sustained gastric release. For example, chitosan–curcumin nanoparticles have demonstrated superior antibacterial and anti-inflammatory activity compared to free curcumin in preclinical models (Abou Baker, 2020; Kamankesh et al., 2024). Such nanocarrier formulations could help overcome pharmacokinetic limitations of natural compounds and enable effective combination with standard antibiotics. A few nutraceutical formulations combining phytochemicals with zinc carnosine, vitamin C, or probiotics have shown preliminary benefits in symptom improvement and partial eradication enhancement. The major phytochemicals evaluated for anti-*H. pylori* activity, along with their sources, mechanisms of action, and key experimental findings, are summarized in Table 2. These findings collectively support that natural bioactives, particularly when optimized through nanocarrier systems or heat-assisted extraction, enhance antibacterial potency while providing antioxidative and anti-inflammatory benefits. While preclinical data are encouraging, clinical trials are needed to validate their efficacy, establish optimal dosing, and to assess synergy with antibiotics. These natural agents are best considered as supportive adjuncts, not replacements, within comprehensive *H. pylori* treatment regimens.

**TABLE 2:** Natural products and active phytochemicals showing inhibitory activity against H. pylori.

Major source/plant	Active component(s)	Mechanism of action	MIC range	References
Thymus kotschyanus	Terpenoid: thymol	Damages membrane integrity, interferes with ATP synthesis, inhibits biofilm production, modulates pH homeostasis	64–128 μg/mL	(Deng et al., 2024)
Origanum vulgare	Terpenoid: carvacrol	Membrane disruption, biofilm and ATP synthesis inhibition	16–64 μg/mL	(Deng et al., 2024)
Pimenta racemosa (stem essential oil)	Terpenoid: eugenol	Urease binding (predicted by docking), bacteriostatic activity, membrane disruption	3.9 μg/mL (stem oil)	(Ayoub et al., 2022)
Glycyrrhiza inflata Batalin (Fabaceae)	Chalcone (Xinjiachalcone A)	Inhibits motility and urease, prevents epithelial interaction	12.5 and 50 μM	(Michalkova et al., 2023; Bodet et al., 2014)
Myrica species	Myricetin	Increases immune detection, antibiotic sensitivity, and disrupts biofilm	160 μg/mL	(Deng et al., 2024)
Cornus canadensis	Tellimagrandin I and II	Damages membrane integrity	6.25–12.5 μg/mL	(Deng et al., 2024)
Curcuma longa (Turmeric)	Curcumin	Urease inhibition, immunomodulation, virulence factor interaction, antioxidant effects	30 μg/mL	(Deng et al., 2024)
Rhizoma Coptidis	Coptisine	Urease inhibition, Cag expression decrease, membrane disruption, DNA fragmentation	21 μg/mL	(Tang et al., 2023)
Hydrastis canadensis	Berberine	Bacteriostatic/bactericidal activity, membrane disruption	0.78 μg/mL	(Salehi et al., 2018)
Tinospora sagittate	Palmatin	Urease inhibition, anti-adhesion to gastric cells	3.12–6.25 μg/mL	(Salehi et al., 2018)
Piper nigrum (Black Pepper)	Piperine	Reduces motility, prevents adhesion to gastric cells	125 μM	(Tharmalingam et al., 2014)
Glycyrrhiza glabra	Glycyrrhetinic acid	Cytotoxic to *H. pylori*, impairs growth	50 μg/mL	(Deng et al., 2024)
Calophyllum brasiliense Cambess.	Brasiliensic and isobrasiliensic acids	Urease inhibition and modulation of anti-inflammatory responses in gastric tissue	31 μg/mL	(do Carmo Souza et al., 2009)
Cuminum cyminum	Cuminaldehyde	Multi-target enzyme inhibition; anti-inflammatory	3.9 μg/mL	(Alomar et al., 2022)
Pimpinella anisum	Anethole	Membrane disruption; bacteriostatic activity	15.63 μg/mL	(Alomar et al., 2022)
Carum carvi	Carvone	Bacteriostatic effects; membrane interference	62.5 μg/mL	(Alomar et al., 2022)
Amomum villosum	Camphor, Borneol, Limonene	Antibiofilm; membrane disruption	256 μg/mL	(Huang et al., 2025)
Salvia officinalis	Carnosic acid	COX-2 inhibition; growth inhibition	3.9–15.63 g/mL	(Alomar et al., 2023)
Haloxylon articulatum	N-Caffeoyltyramine	Isoleucyl-tRNA synthetase inhibition	54 μg/mL	(Al-Rashidi et al., 2025)
Haloxylon articulatum	Sinapoyltyramine	Isoleucyl-tRNA synthetase inhibition	74 μg/mL	(Al-Rashidi et al., 2025)
Allium sativum.	Diallyl tetrasulfide and allicin	Urease inhibition, membrane disruption, interference with thiol enzymes, reduced motility and adhesion	3–6 μg/mL and 4 μg/mL	(Salehi et al., 2018)
Glycyrrhiza glabra (licorice)	Flavonoid mixture (GutGard^®^)	Possible inhibiting protein synthesis, DNA gyrase and dihydrofolate reductase	32 and 100 μg/mL	(Asha et al., 2013)
Multi-herb: Terminalia chebula, Ficus hirta, Syzygium aromaticum	Complex phytochemical mixture: Hezi Qingyou Formula (HZQYF)	Downregulation of adhesion/urease genes, increased membrane permeability, decreased urease activity	80–320 μg/mL	(Feng et al., 2024)
Sanguisorba officinalis	Multi-component (polyphenols, gallic acid and ellagic acid)	Multi-target effects: cell wall disruption, morphological alterations, metabolic pathway interference and transcriptional changes	80–320 μg/mL (flower extract)	(Chen et al., 2024)

##### 5.3.2.4 Vaccines and host-directed strategies

Immunomodulatory therapies like Licopid (a muramyl dipeptide derived from *Lactobacillus bulgaricus*) and various nutraceuticals have shown modest improvements in *H. pylori* eradication rates and may help reduce reinfection risk by enhancing mucosal immunity (Konorev et al., 2016). Vaccine development remains challenging due to the genetic diversity and immune evasion strategies of *H. pylori*. Although whole-cell, subunit, DNA, and live vector vaccines have been explored, none have achieved more than 50% efficacy in phase III trials (Sutton and Boag, 2019). Among the most promising candidates are subunit and multi-epitope vaccines targeting conserved bacterial antigens such as CagA, VacA, BabA, SabA, HopQ, OipA, Hsp60, and NapA. Therapeutic vaccines incorporating recombinant CagA and VacA have demonstrated the ability to clear chronic infections in murine models (Sedarat and Taylor-Robinson, 2024; Keikha et al., 2019). Subunit vaccines like recombinant urease, one of the earliest candidates, showed up to 70% short-term efficacy, though protection declined over time. Fusion vaccines such as FliD–UreB–CagL–VacA and multi-antigen formulations like CTB-multiHp have elicited strong mucosal and systemic immune responses in preclinical models (Ghasemi et al., 2018). Recent advances include the use of computational and AI-driven methods to design multi-epitope vaccines with broader immunogenicity, but these require further validation. Host-directed strategies, including immunomodulation such as boosting Th1/Th17 responses or regulating T cell function, microbiome modulation through probiotics or dietary interventions to create a more resilient host environment, and enhancement of gastric mucosal defenses, are being studied as adjuncts or alternatives to antibiotic therapy (Tu et al., 2024). Despite encouraging findings, these therapies require further standardization, large-scale validation, and optimization of delivery strategies before they can be widely integrated into clinical practice.

##### 5.3.2.5 Nanoparticle delivery systems

Nanoparticle-based delivery systems are emerging as a powerful tool in *H. pylori* eradication by enhancing drug stability, mucosal targeting, and therapeutic synergy. Poly(lactic-co-glycolic acid) (PLGA) nanoparticles, such as AMX-PLGA/UCCs-2, protect against gastric degradation and release the drug at infection sites, enhancing efficacy and reducing toxicity (Luo et al., 2018). Lipid-based systems like liposomes loaded with linolenic acid (LipoLLA) and pectin-coated liposomes further improve mucosal adhesion and can disrupt *H. pylori* membranes, including antibiotic-tolerant coccoid forms (Thamphiwatana et al., 2014). Silver nanoparticles (AgNPs) in combination with probiotics like *L. plantarum* showed synergistic effects by disrupting bacterial biofilms, reducing antibiotic resistance, and promoting gastric healing (Vijayakumar et al., 2023). Despite promising preclinical outcomes including up to 99% bacterial inhibition and 96% wound closure, widespread application of these agents is currently limited by manufacturing complexity and the need for further safety and clinical validation.

Although not all studies met the inclusion criteria for detailed tabulation due to incomplete reporting of adverse effects or resistance-specific efficacy, they collectively reinforce key themes in current *H. pylori* research: the importance of tailoring regimens to local resistance trends, the potential of adjunctive therapies to improve compliance, and the need for broader integration of resistance diagnostics in clinical decision-making.

## 6 Conclusion and future perspectives

The management of *Helicobacter pylori* infection is undergoing significant transformation due to increasing antibiotic resistance, declining eradication rates with older regimens, and the growing need for individualized treatment strategies. Data from recent clinical trials have consistently demonstrated the limited effectiveness of STT, particularly in regions where clarithromycin resistance exceeds > 20%, significantly compromising treatment success. In contrast, quadruple regimens that include bismuth or the novel acid suppressants have shown superior efficacy and improved tolerability. Among these, the novel P-CAB, vonoprazan, with its high efficacy even in resistant settings has emerged as a promising alternative to conventional PPIs, and growing support in updated treatment guidelines. In addition to antibiotic-based therapies, adjunctive approaches using probiotics, phytochemicals, and phage-derived enzymes have gained attention for their potential to enhance treatment outcomes and reduce gastrointestinal side effects. The incorporation of resistance-guided therapy and molecular diagnostics into clinical practice also offers opportunities to tailor regimens more precisely based on local resistance patterns and individual patient characteristics. However, several challenges remain. Reinfection is still common in high-prevalence settings, and access to susceptibility testing is limited in many low-resource regions. Furthermore, resistance to second-line and salvage therapies, including levofloxacin and rifabutin, is an emerging concern that may compromise future treatment options. To address these challenges, future strategies should emphasize global surveillance of antimicrobial resistance, broader implementation of rapid molecular diagnostic tools, and the development of innovative non-antibiotic therapies. There is also a pressing need for continued evaluation of vaccine candidates to prevent initial infection and reinfection, as well as the expansion of screen-and-treat programs in high-incidence populations. A comprehensive approach that integrates therapeutic advances, resistance monitoring, and public health initiatives will be essential to reduce the global burden of *H. pylori*-associated diseases.
